# Traumatic brain injury in forensic psychiatry

**DOI:** 10.1192/j.eurpsy.2021.1895

**Published:** 2021-08-13

**Authors:** J. Regala, P. Ferreira, F. Vieira

**Affiliations:** Forensic Psychiatry Department, Centro Hospitalar Psiquiátrico de Lisboa, Lisboa, Portugal

**Keywords:** traumatic brain injury, neuropsychiatric sequelae, civil litigation, diffuse axonal injury

## Abstract

**Introduction:**

Assessment of neuropsychiatric sequelae of traumatic brain injury (TBI) brings about challenges in the forensic setting, comprising analysis of neurobiological variables, preinjury variables (personality/psychiatric disturbances), postinjury psychosocial, allowing the expert witness to provide clear and appropriate explanations, so the court can decide with justice, particularly in civil law cases.

**Objectives:**

Discuss the main clinical and neuroimagiologic aspects to consider in civil litigation of TBI cases.

**Methods:**

Comprehensive literature review.

**Results:**

Although accurate predictions are difficult, some generalizations can be made. Recovery from hypoxic and diffuse axonal injury (DAI) takes longer and is less complete than focal contusions. Posttraumatic amnesia is the main predictor of long-term cognitive outcome. In moderate/severe TBI (m/sTBI) occurs chronic lesion expansion (axonal degeneration) and brain atrophy. DAI topography determinates the cognitive disfunction pattern yet underestimated in conventional neuroimaging. Diffusion-Tension-Imaging (DTI) may be valuable to outcome predictions in m/sTBI: structural disconnection within the Default Mode and the Salience Networks are linked to attention and executive impairments; hippocampus and fornix damage correlates with memory/learning impairments. Conversely, DTI findings can be misleading in mild TBI (mTBI), and case-by-case analysis seldomly prove its scientific validity.

**Conclusions:**

To elaborate formulations within reasonable medical certainty, outcome predictions should not be made until at least six months following the TBI, considering that most mTBI symptoms resolve in few months, and up to 1-½ years, when m/sTBI neuropathologic changes stabilize. The neurobiological underpinnings are fundamental for causality formulations, however atypical outcomes in mTBI are frequently predicated upon non–brain-injury psychiatric conditions and psychosocial factors.

**Disclosure:**

No significant relationships.

